# Assessment of Imputation Quality: Comparison of Phasing and Imputation Algorithms in Real Data

**DOI:** 10.3389/fgene.2021.724037

**Published:** 2021-09-22

**Authors:** Katharina Stahl, Damian Gola, Inke R. König

**Affiliations:** ^1^Department of Genetic Epidemiology, University Medical Center, University of Göttingen, Göttingen, Germany; ^2^Institut für Medizinische Biometrie und Statistik, Universität zu Lübeck, Universitätsklinikum Schleswig-Holstein, Lübeck, Germany; ^3^German Center for Cardiovascular Research, Partner Site Hamburg/Kiel/Lübeck, Lübeck, Germany

**Keywords:** imputation, phasing, accuracy, quality, speed, DZHK, HRC

## Abstract

Despite the widespread use of genotype imputation tools and the availability of different approaches, late developments of currently used programs have not been compared comprehensively. We therefore assessed the performance of 35 combinations of phasing and imputation programs, including versions of SHAPEIT, Eagle, Beagle, minimac, PBWT, and IMPUTE, for genetic imputation of completely missing SNPs with a HRC reference panel regarding quality and speed. We used a data set comprising 1,149 fully sequenced individuals from the German population, subsetting the SNPs to approximate the Illumina Infinium-Omni5 array. Five hundred fifty-three thousand two hundred and thirty-four SNPs across two selected chromosomes were utilized for comparison between imputed and sequenced genotypes. We found that all tested programs with the exception of PBWT impute genotypes with very high accuracy (mean error rate < 0.005). PBTW hardly ever imputes the less frequent allele correctly (mean concordance for genotypes including the minor allele <0.0002). For all programs, imputation accuracy drops for rare alleles with a frequency <0.05. Even though overall concordance is high, concordance drops with genotype probability, indicating that low genotype probabilities are rare. The mean concordance of SNPs with a genotype probability <95% drops below 0.9, at which point disregarding imputed genotypes might prove favorable. For fast and accurate imputation, a combination of Eagle2.4.1 using a reference panel for phasing and Beagle5.1 for imputation performs best. Replacing Beagle5.1 with minimac3, minimac4, Beagle4.1, or IMPUTE4 results in a small gain in accuracy at a high cost of speed.

## 1. Introduction

In typical large-scale genetic association studies, the participants are genotyped using commercially available genotyping arrays to measure genetic variants across the entire genome. However, these arrays only type up to 4 million variants, depending on the specific version and producer of the array. Comparing this with the more than 84 million single nucleotide polymorphisms (SNPs) in the human genome identified by the The 1000 Genomes Project Consortium (2015), the typically typed variants therefore only comprise a fraction of the known variants. This is a cost-efficient strategy, given that more than 90% of known SNPs are highly correlated with at least one typed variant (Li et al., 2008; Ha et al., 2014).

However, for many applications, it is desirable to fill in the genotypes of the untyped variants. For example, meta-analyses of association studies that used different genotyping arrays are hampered by the fact that different numbers and selections of SNPs are available from the different arrays (Anderson et al., 2008; Marchini and Howie, 2010). A meta-analysis would therefore lose power due to a possibly small overlap of SNPs. For this, genotype imputation is used to estimate missing SNPs with a reference panel appropriate for the population under investigation. It is thus possible to fill in both randomly missing genotypes from SNPs which were typed on the array but missing in some probands, and SNPs which were not part of the array but present in the reference panel. There are a number of further advantages to the use of an imputed genotype data set (Li et al., 2009). First, using a data set containing more SNPs leads to more power in genome-wide association studies and similar analyses, because the pool of SNPs and the genetic variety is larger (Marchini and Howie, 2010; Pei et al., 2010). Second, more SNPs lead to a higher resolution and thus help to localize areas of interest in the genome. Third, it is possible that the imputed SNPs are closer to the disease-causing variant than the genotyped SNPs and therefore are able to pick up signals that might have been missed otherwise (Orho-Melander et al., 2008).

The principle of imputation in general is to leverage linkage disequilibrium to identify shared DNA sequences between the target data and the reference data from a common ancestor. With these shared sequences, the missing genotypes are inferred from the reference panel using different methods. This implies that the reference panel and the target data set should stem from the same ethnic population for the imputation to yield accurate results. The imputation procedure is usually divided into two steps: First, given genotype data without haplotypic information, the data needs to be phased first to deduce haplotypes. These estimated haplotyes are then used in the second step to impute missing genotypes. Although this separation of the two steps leads to a small loss in accuracy (Roshyara et al., 2016), it is common practice for computational efficiency and ability to handle large data sets.

Several imputation algorithms have been developed and implemented including different versions of IMPUTE (Howie et al., 2009, 2011; Bycroft et al., 2017), minimac (Das et al., 2016), Beagle (Browning and Browning, 2016; Browning et al., 2018) and the positional Burrows-Wheeler transform (PBWT) (Durbin, 2014), and a recent review on the role of genotype imputation in genome-wide association studies was given by Naj (2019). In addition to software that can be installed and run on local computers, imputation servers offer automated remote phasing and imputation pipelines, for example the Sanger Imputation Service^1^. The quality of the imputation has been assessed several times with different focuses using a subset of the now available programs. Liu et al. (2015) used a subset of fully sequenced real data to compare the performance of pairs of phasing and imputation protocols and assessed the quality by comparison with the originally sequenced genotypes. Of note, they only considered pairs of phasing and imputation tools developed by the same research group. Das et al. (2016) found that with larger reference panels, imputation accuracy increases. Browning et al. (2018) assessed the imputation accuracy of fairly recent developments with simulated data, finding no large differences between the used programs in terms of imputation quality. Shi et al. (2018) used a small fully sequenced sample from the Chinese population to evaluate several aspects of imputation quality, including the effect of sequencing coverage, sample size and SNP density and MAF, showing among other results, that imputation accuracy is low in rare variants. Schurz et al. (2019) compared the imputation quality of the current imputation servers to the same workflow on their own servers in the highly admixed South African population, finding that a remote PBWT-based imputation yielded the best results.

However, late developments in imputation and phasing tools have not been assessed yet.

We therefore compared the performance of 35 combinations of phasing and imputation procedures including versions of SHAPEIT (Delaneau et al., 2013), Eagle (Loh et al., 2016a,b), Beagle (Browning and Browning, 2016; Browning et al., 2018), minimac (Das et al., 2016), PBWT (Durbin, 2014), and IMPUTE (Howie et al., 2009, 2011; Bycroft et al., 2017) based on real sequenced data. With a subset of a fully sequenced data set from the German population, we emulated the use of a common SNP array and then imputed completely missing SNPs, thus allowing for a direct comparison of sequenced and imputed genotypes. To investigate the advance of the versions, we also included still frequently used older versions of the imputation programs. In this analysis, we focus on in-house imputation only, since not all data can be uploaded to remote servers for reasons of security or confidentiality. We assessed the imputation quality using a wide variety of quality measures, including scores that leverage the known, true underlying genotype, such as the Hellinger score (Roshyara et al., 2014), and scores which are more commonly used to estimate the imputation quality based on the estimated genotypes like the Beagle *R*^2^ (Browning and Browning, 2009). We also investigated the imputation quality depending on the minor allele frequency (MAF) and the genotype probability of the imputed genotypes.

## 2. Materials and Methods

### 2.1. Data

We used a data set from the German Centre of Cardiovascular Research (DZHK) comprising whole genome sequence data from 1,149 healthy controls of the German population^2^. Individuals were recruited in six centers in Germany. Participating cohorts are the Gutenberg-Gesundheitsstudie^3^, the Hamburg City Health Study^4^, the Heidelberg Normal Kontrollen (NOKO) of the University Heidelberg, the project KORA by the HelmholtzZentrum München^5^, the Study of Health in Pomerania^6^ and the resources of the Institut for Molekularbiologie Kiel^7^. To avoid systematic differences between the cohorts, the centers used the same standard operatic procedures, and all sequencing was performed on the HiSeq-X platform of the High Throughput Sequencing Unit of the Deutsches Krebsforschungszentrum (German center of cancer research) in Heidelberg. The data was processed at the University Lübeck and the HelmholtzZentrum München. The average sequence depth is 37.75 with at least 30 for each sample. The cutoff for single-nucleotide variants in the VQSR analysis is 99.8%. More details on the data set is reported elsewhere (Berutti et al., 2020).

To emulate Illumina's Infinium Omni5 array for genotyping on chromosomes 19 and 22, a subset of 114,487 SNPs was extracted from this data. Individuals with more than 10% of genotypes missing and SNPs that did not pass the quality control conducted by the variant quality score recalibration (VQSR) included in the Genome Analysis Toolkit (GATK)^8^ were removed before further analysis, so the data was further reduced to 109,874 SNPs.

For both imputation and phasing, we used a reference panel provided by the Haplotype Reference Consortium (HRC) comprising 27,165 individuals.

### 2.2. Phasing and Imputation

**Figure 1:**
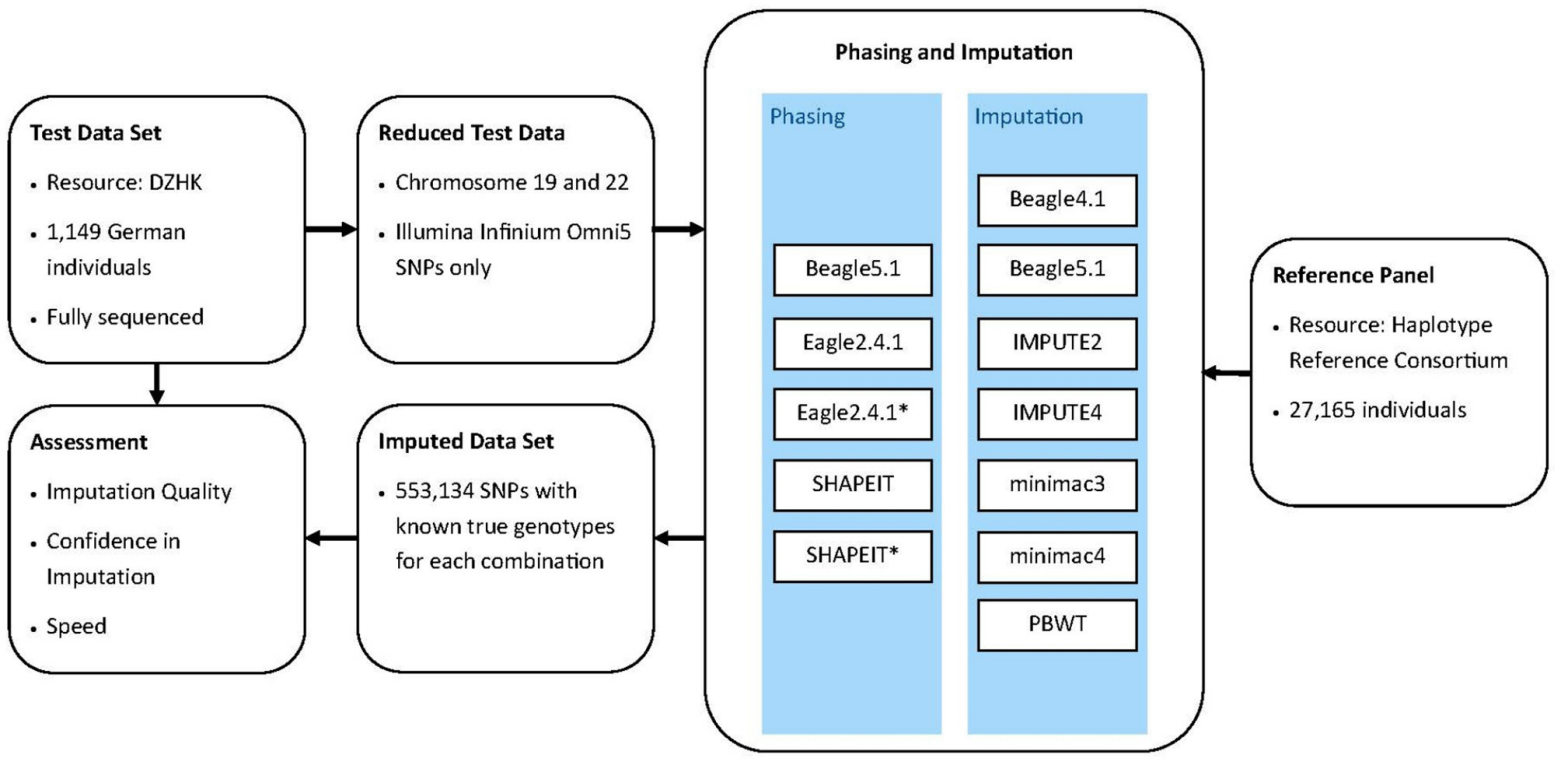
Workflow for imputation quality assessment. Phasing tools marked with an asterix indicate the use of a reference panel for phasing.

The workflow of this analyis is depicted in Figure 1. We began the imputation and phasing processes in October 2019 and finished in January 2020. Starting from the data set of SNPs emulating the use of Illumina's Infinium Omni5 array for genotyping, we imputed missing SNPs using the following programs: IMPUTE2 (Howie et al., 2009, 2011), IMPUTE4 (Bycroft et al., 2017), minimac3 (Das et al., 2016), minimac4, Beagle4.1 (Browning and Browning, 2016), Beagle5.1 (Browning et al., 2018) and PBWT (Durbin, 2014). Phasing was conducted with either Eagle2.4.1 (Loh et al., 2016a,b), Beagle5.1 (Browning and Browning, 2007) or SHAPEIT2 (Delaneau et al., 2013). The online resources of those tools can be found in Table 1. Eagle2.4.1 and SHAPEIT2 allow phasing with a reference panel, so we conducted the phasing step with and without the reference data for both programs. The seven imputation tools and the five phasing varieties add up to 35 combinations of phasing and imputation protocols. A later version of SHAPEIT is available, but was not considered, because SHAPEIT3 is not recommended for data sets of our sample size.

**Table 1:** Online resources for the phasing and imputation tools considered.

**Tool**	**Online resource**
Beagle4.1	https://faculty.washington.edu/browning/beagle/b4_1.html
Beagle5.1	https://faculty.washington.edu/browning/beagle/beagle.html
Eagle2	https://data.broadinstitute.org/alkesgroup/Eagle/
Impute2	https://mathgen.stats.ox.ac.uk/impute/impute_v2.html
Impute4	https://jmarchini.org/software/
minimac3	https://genome.sph.umich.edu/wiki/Minimac3
minimac4	https://genome.sph.umich.edu/wiki/Minimac4
PBWT	https://github.com/richarddurbin/pbwt
Shapeit2	https://mathgen.stats.ox.ac.uk/genetics_software/shapeit/shapeit.html

With the exception of PBWT, the programs use a variation of Hidden Markov Models (HMMs) to infer haplotypes for phasing and to infer missing genotypes for imputation. The general idea in the imputation process is to use the haplotypes of the reference data set as the hidden states for the observed target data set and to mimick recombination events with the probabilities defining the HMM (Li and Stephens, 2003). In contrast, PBWT uses the positional Burrows-Wheeler transformation to locate shared sequences between the target data set and the reference panel and infer missing genotypes by overlapping shared sequences (Durbin, 2014). Details on the implemented algorithm are not published yet.

Before phasing and imputation, the reference panel was converted into the .haps/.legend format for SHAPEIT2, IMPUTE2, and IMPUTE4 as well as into the VCF format for minimac3 and Beagle4.1. For PBWT, Beagle 5.1 and minimac4, the reference data had to be transformed into program specific formats.

The phasing was conducted separately for chromosomes 19 and 22, resulting in five phased data sets for each chromosome, one phased with Beagle5.1 and two phased with SHAPEIT2 and Eagle2.4.1 each. To combine every phasing protocol with every imputation tool, we formated the phased data set accordingly, if necessary. With the exception of PBWT, IMPUTE2, and IMPUTE4, the imputation programs were able to use the output of the phasing programs in the VCF format. While SHAPEIT2 already provides files in the .haps/.sample format as an output, we used BCFtools^9^ to convert the output formats of the other phasing programs into a .haps file for IMPUTE2 and IMPUTE4. PBWT seems only to impute SNPs that are explicitly marked as missing, so we added the SNPs of the reference panels as missing SNPs into the VCF-file before imputation.

We then imputed the missing genotypes in chunks of about 5Mbp with the exception of PBWT, because PBWT does not support chunking. This translates to 21 chunks, namely 13 on chromosome 19 and 8 on chromosome 22, to parallelize the imputation processes where possible. For computational reasons, we had to divide the data set for the imputation on chromosome 19 with PBWT into two data sets of 574 and 575 individuals, which were later merged into one data set again. The phasing and imputation process was parallelized using the R package batchtools (Bischl et al., 2015; Lang et al., 2017; R Core Team, 2020). The R code we employed is publicly available^10^.

As output formats, we derived both dosages and best-guess genotypes from the genotype probabilities.

### 2.3. Quality Measures

To estimate the accuracy of imputation in terms of comparing imputed with sequenced genotypes, we used the concordance rate, the Imputation Quality Score (IQS) (Lin et al., 2010), the Hellinger score (Roshyara et al., 2014), and the squared Euclidean norm score (SEN score) (Roshyara et al., 2014). The concordance rate is calculated as the proportion of correctly imputed best-guess genotypes of all imputed genotypes. The IQS is a concordance rate adjusted for chance with a maximum score of 1 and no theoretical minimum (Lin et al., 2010), while 0 indicates that assigning genotypes randomly according to the true allele frequencies would yield the same proportion of correctly imputed best-guess genotypes. Both concordance rates use the best-guess genotypes and are calculated for every SNP separately. The raw concordance was also converted into an error rate.

In contrast, the Hellinger score uses the genotype probability. It is based on the Hellinger distance (Roshyara et al., 2014) and ranges from 0 to 1, with a score close to 1 indicating a high similarity between the distribution of the imputed genotype probabilities and the true genotype. Since the score is calculated for every imputed SNP for every individual, it then needs to be accumulated across the individuals per SNP. To avoid losing too much information, we extracted for every SNP the mean, the standard deviation (SD), the minimum, the maximum, the median and the quartiles. Thus, the minimum of the Hellinger score may be interpreted as the worst-case imputation quality.

Finally, the SEN score is based on the dosage and summarizes the distance between the imputed dosage and the true genotype (Roshyara et al., 2014). Similar to the Hellinger score, it is calculated per every individual SNP, ranges between 0 and 1, and higher scores indicates a higher imputation quality. Again, it is accumulated across the individuals.

All of the above measures allow for a comparison with the ground-truth of sequenced genotypes. However, in usual applications, these are unknown, hence the imputation in the first place. We therefore included a second category of quality measures which estimate the imputation quality without using the sequenced genotypes, namely the MaCH *R*^2^, the Beagle *R*^2^ and the IMPUTE Info score. These scores provide more insight into how confident the imputation program is in the estimated genotype rather than true imputation quality in the sense of concordance with the true genotypes. As the names already suggest, the scores are implemented in the minimac, Beagle and IMPUTE imputation programs, respectively. For a better overview, we calculated these for all imputed genotypes regardless of the programs used, even though they are highly correlated.

The MaCH *R*^2^ estimates the ratio between the observed variance of the imputed genotypes and the expected variance if the population was in Hardy-Weinberg equilibrium (Marchini and Howie, 2010). It approximates the correlation between the dosage and the true genotype. The Beagle *R*^2^ is closely related to the MaCH *R*^2^ and approximates the squared correlation between the best guess genotype and the true genotype (Browning and Browning, 2009). The IMPUTE Info score estimates the ratio between the observed and expected statistical information (Marchini and Howie, 2010). All these measures depend on the estimated MAF of the imputed genotypes, resulting in difficulties of detecting incorrectly imputed genotypes if the imputation suggests a monomorphic SNP.

To gain insight into the imputation quality in practical settings, we assessed the concordance depending on the genotype probabilities. For this purpose, the concordance rate was calculated on individually imputed genotypes within a range of genotype probabilities for each SNP, which was then averaged. Finally, we assessed the concordance for different MAFs in minor allele genotypes, which include heterozygous and alternative allele homozygous genotypes, discussing possible cutoffs for MAF and gentoype probabilities to exclude imputed SNPs which might impair the overall imputation quality.

## 3. Results

With each program combination 1,389,448 SNPs were imputed. The overlap between imputed SNPs and SNPs contained in the original DZHK data set consists of 553,234 SNPs, which we used to assess the imputation quality. The difference in numbers between the imputed and overlapping SNPs is most likely caused by differences in coverage between the reference panel and the original data set.

**Figure 2:**
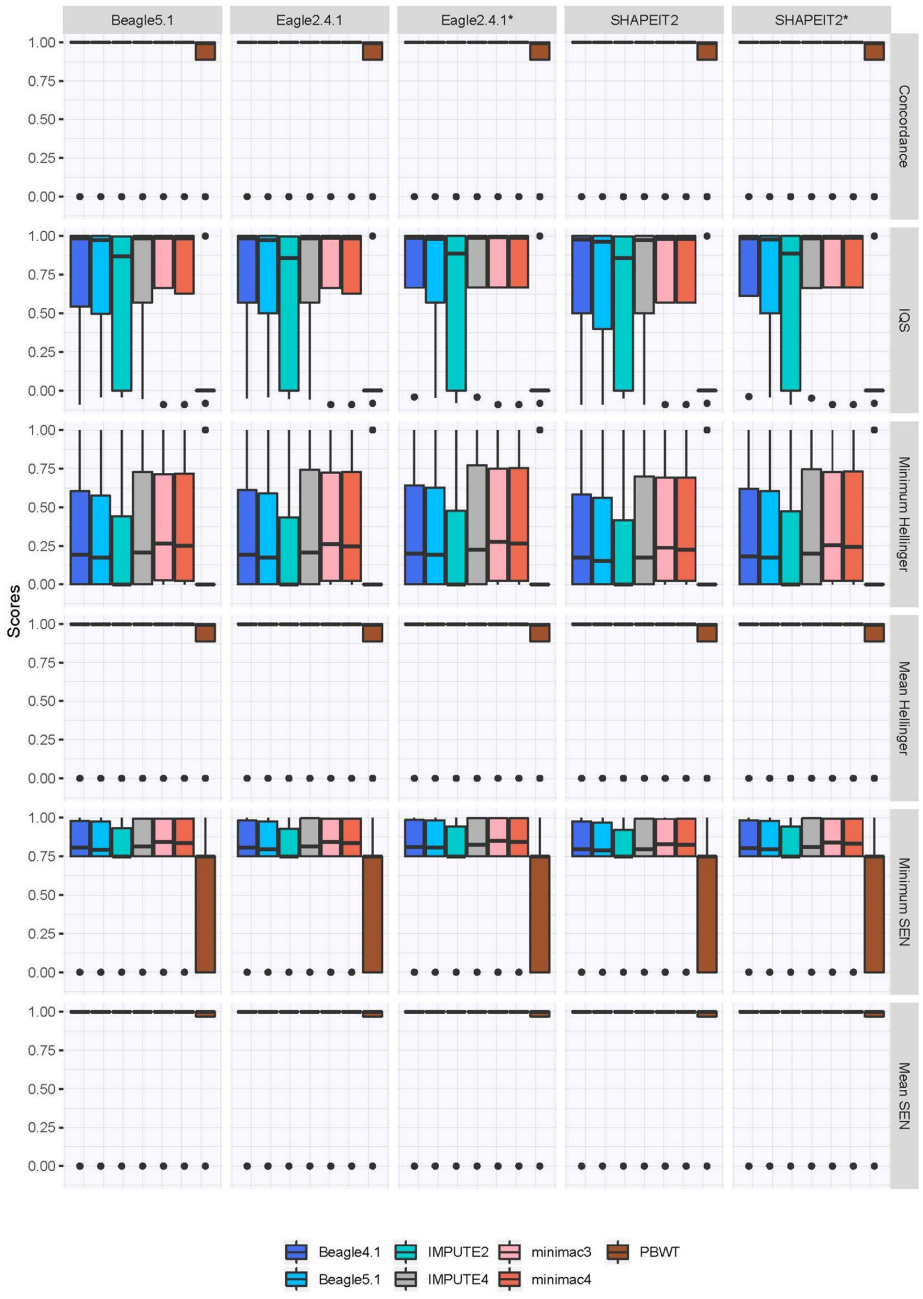
Imputation quality measures based on sequenced genotype for comparison. For the Hellinger and the SEN score, the minimum and mean are included. Phasing tools marked with an asterix indicate the use of a reference panel for phasing.

As depicted in Figure 2, the overall imputation quality is very high for most of the imputation and phasing combinations, indicated by the concordance rate, the IQS, the mean Hellinger score and the mean SEN score. PBWT is the only imputation program which yields notably worse results, whereas Beagle4.1, Beagle5.1, IMPUTE4, minimac3, and minimac4 differ only slightly in imputation quality. For a practical overview, Table 2 compiles the error rates in detail. Again, using PBWT or IMPUTE2 for imputation yields a higher error rate compared to the other programs, although the difference between PBWT and the other protocols is more pronounced. The lack of difference between the median error rates of most of the imputation programs indicates that for practical purposes, the performance of the programs is of very similar quality. In addition, the effect of different phasing approaches is only small. As shown in Table 2, the greatest reduction in error is obtained by using Eagle2.4.1 for phasing without an additional reference panel, but, again, the difference is only small. Notable, with the exception of IMPUTE, the newer versions of the programs do not yield better results for imputation quality in our dataset. According to the concordance, the IQS, the minimum and the mean of the SEN-score, and the minimum of the Hellinger score, minimac3 is the best imputation program with a mean error rate of 0.0031401. IMPUTE4 scores highest in the mean of the Hellinger score, which means that IMPUTE4 assigns genotype probabilities more accurately than the other programs. Detailed overviews of all extracted characteristics of the distributions for both the Hellinger score and the SEN score can be found in the Supplementary Material.

**Table 2:** Error rates as mean across phasing protocols and deviation from mean for every phasing protocol.

	**Mean**			**Deviation**		
	**All phasing**	**Beagle5.1**	**Eagle2.4.1**	Eagle2.4.1^*^	**SHAPEIT2**	SHAPEIT2^*^
Beagle4.1	0.0033016	3.0357 × 10^−5^	−1.2997 × 10^−5^	8.2931 × 10^−5^	−9.7560 × 10^−5^	-2.7316 × 10^−6^
Beagle5.1	0.0033571	2.7450 × 10^−5^	−6.6318 × 10^−6^	6.8414 × 10^−5^	−9.9092 × 10^−5^	9.8597 × 10^−6^
IMPUTE2	0.0042061	4.2378 × 10^−6^	−4.7412 × 10^−5^	9.7701 × 10^−5^	−9.0420 × 10^−5^	3.5893 × 10^−5^
IMPUTE4	0.0033572	3.2415 × 10^−5^	−2.6751 × 10^−5^	1.0125 × 10^−4^	−1.1219 × 10^−4^	5.2721 × 10^−6^
minimac3	0.0031401	3.0646 × 10^−5^	−1.7680 × 10^−5^	8.6474 × 10^−5^	−9.7262 × 10^−5^	−2.1784 × 10^−6^
minimac4	0.0031904	3.0353 × 10^−5^	−1.7605 × 10^−5^	8.8464 × 10^−5^	−1.0018 × 10^−4^	−1.0363 × 10^−6^
PBWT	0.1354054	1.6959 × 10^−7^	1.6959 × 10^−7^	1.6959 × 10^−7^	1.6801 × 10^−7^	−6.7678 × 10^−7^

*Phasing tools marked with an asterix indicate the use of a reference panel for phasing. The median is not included because it has the same value of 0.00087 for every combination of phasing and imputation with the exception of PBWT, which has a median error rate of 0.00522 for all phasing protocols*.

**Figure 3:**
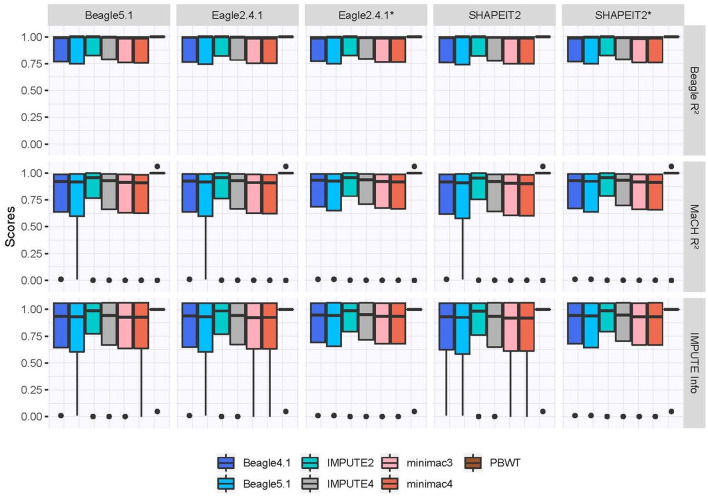
Imputation quality measures usable without knowledge of true genotypes. Phasing tools marked with an asterix indicate the use of a reference panel for phasing.

The quality measures in the second category assessing the confidence in the imputation without knowing the true genotypes are shown in Figure 3 and rank the programs differently. While MaCH *R*^2^, Beagle *R*^2^ and the IMPUTE Info score recognize that the imputation quality in general is high, the programs with low scores in Figure 2 are estimated to have the best imputation quality with PBWT as the program with the highest confidence in the imputation.

**Figure 4:**
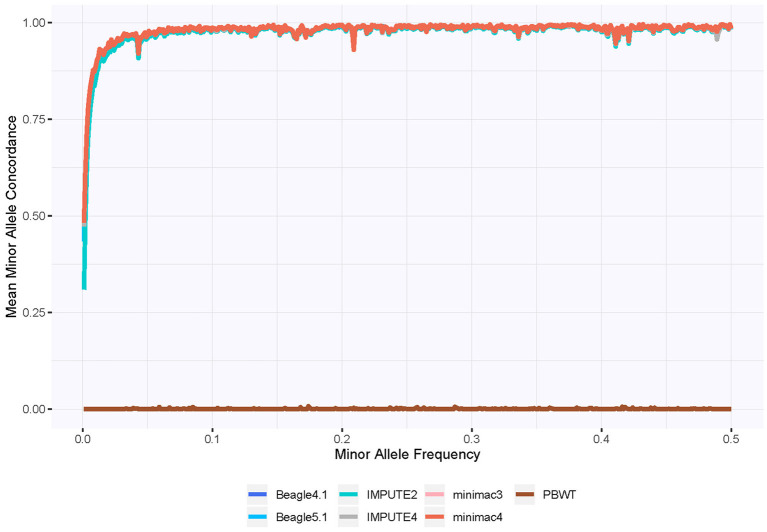
Concordance rate for imputed genotypes with true genotypes not homozygous with the reference allele, depending on MAF. Beagle 4.1, Beagle 5.1 Impute4, minimac3, and minimac4 almost perfectly align. Different phasing processes were not considered. For a detailed view of the region with MAF < 0.05, see the Supplementary Material.

The concordance of the minor allele genotypes depending on the MAF is depicted in Figure 4. We excluded genotypes that would be correctly imputed as homozygous with the reference allele, because we want to investigate at which point rare variants are not accurately imputed anymore. This is necessary, because overall concordance would still be high for monomorphic imputed SNPs, if their MAF is low enough. We averaged the concordance of those minor allele genotypes according to their MAFs, using MAF intervals of 0.001 for the grouping to ensure a both detailed and clear performance overview. Beagle4.1, Beagle5.1, IMPUTE4, minimac3 and minimac4 impute the rare variants slightly better than IMPUTE2, while PBWT tends to choose the reference allele most of the time in the imputation process regardless of the MAF. In general, the concordance starts to decline for all imputation programs for genotypes involving the minor allele if the MAF is below 0.05. For rare variants with a MAF below 0.01, the concordance drops drastically. A detailed view of the area with MAF < 0.05 is included in the Supplementary Material.

**Figure 5:**
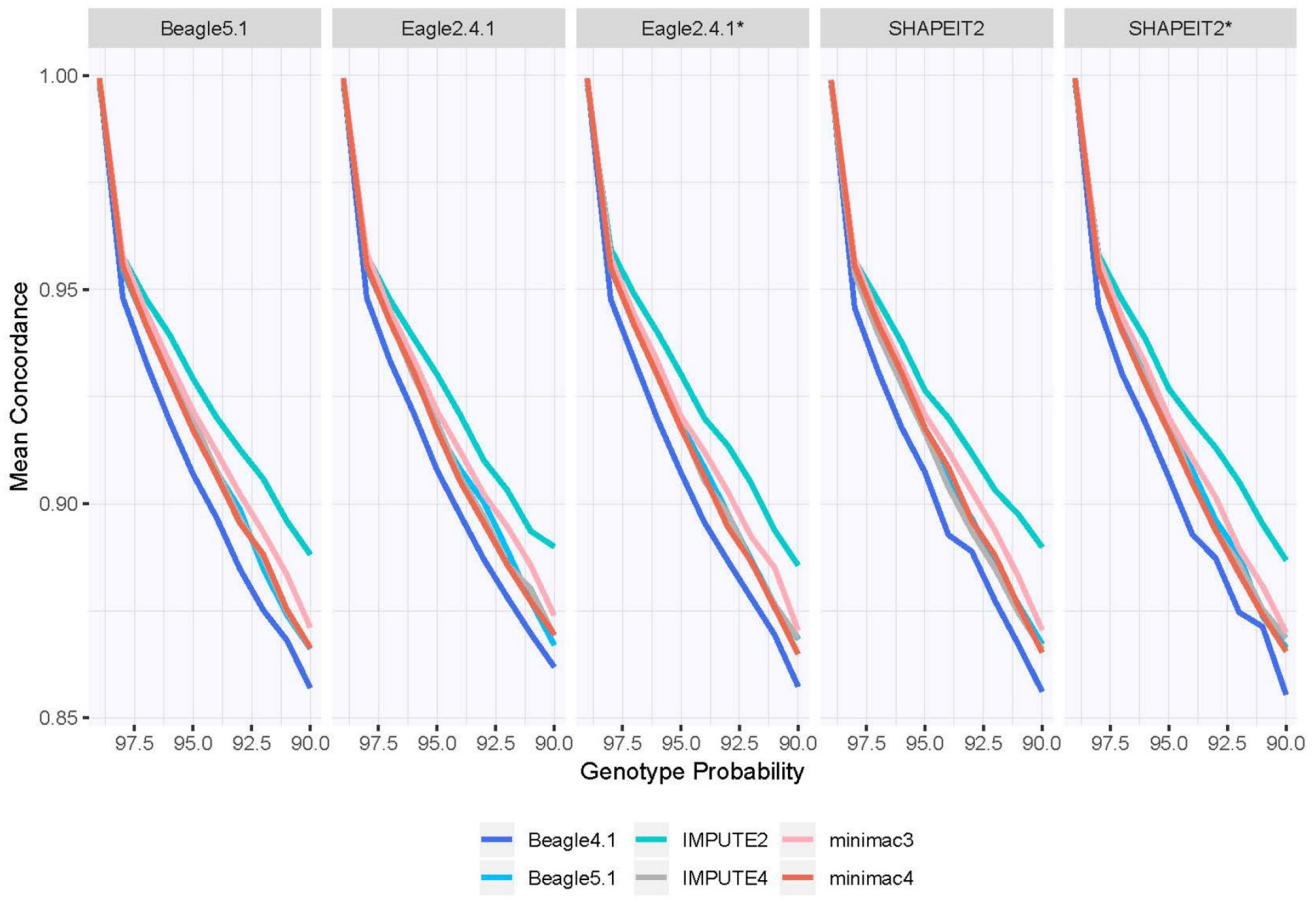
Concordance rate of imputed best-guess genotype depending on genotype probability. The interval length of genotype probabilities was set to 0.01 with the lower border fixed to the x axis. PBWT is not included in this graphic because of its low values. For a broader view including PBWT, see the Supplementary Material. Phasing tools marked with an asterix indicate the use of a reference panel for phasing.

Figure 5 shows the concordance rate depending on the genotype probability of the best-guess genotype. Similar to Figure 4, genotypes were grouped using intervals of genotype probabilities with the length of 0.01. The concordance of PBWT is generally so low that it is not included but given in the Supplementary Material. For all other imputation programs the concordance drops quickly with lower genotype probabilities with the gradient being most and least severe for Beagle4.1 and IMPUTE2, respectively.

In response to the question when to discard imputed genotypes for practical use, Table 3 lists thresholds for MAF and genotype probabilities for each of the imputation programs that still maintain an acceptable level of concordance. The MAF thresholds were derived with the minor allele concordance. These can be seen as cutoffs to ensure high concordance. PBWT was excluded from Table 3, because it does not meet the required level of concordance overall. Because of the only small influence of phasing, Table 3 depicts the average over the variations in phasing. deleted statement: A complete overview including phasing is given in the supplement. It should be noted that we excluded the local minimum in concordance for a MAF around 0.04, which is depicted in Figure 4. The MAF cutoff is 0.03 for a concordance level of 95% in the minor allele genotypes for IMPUTE2, while Beagle4.1, Beagle5.1, IMPUTE4, minimac3 and minimac3 hold the same level of concordance with slightly rarer SNPs still. For the practical use, this means that if the mean condordance of the minor allele genotypes is to be kept above 95%, SNPs imputed with IMPUTE2 and with a known MAF lower than 0.03 should be discarded. The worse results for IMPUTE2 translate to the lower concordance levels in Table 3 as well. For all imputation programs lower MAFs can be included, if the intended level of minor allele concordance is lowered as well. Beagle4.1, IMPUTE4, minimac3, and minimac4 impute minor allele genotypes with a mean concordance of at least 75%, if the MAF is 0.003 or higher, so the drop in the concordance is not as steep as the drop in resulting MAF cutoff. As already shown in Figure 5, the concordance drops rapidly with lowering genotype probabilities for all genotypes. The genotype probability cutoff for a 95% concordance level for Beagle5.1, IMPUTE2, IMPUTE2, IMPUTE4, minimac3, and minimac4 is 0.98, while for Beagle5.1 the cutoff is 0.99 for the same concordance level. For the 90% concordance level, IMPUTE2 has lowest cutoff with 0.92. Note, that the intervals for genotype probabilities were chosen with a length of 0.01 to ensure clean cutoff values for practical use.

**Table 3:** Cutoffs for MAF in imputed minor allele genotypes and for genotype probabilities (GP) in all genotypes to ensure a level of mean concordance.

	**Cutoff MAF (minor allele genotypes)**					**Cutoff GP**	
**Concordance levels:**	**95%**	**90%**	**85%**	**80%**	**75%**	**95%**	**90%**
Beagle4.1	0.0262	0.0102	0.0062	0.0042	0.0030	0.990	0.950
Beagle5.1	0.0276	0.0108	0.0070	0.0050	0.0032	0.980	0.938
IMPUTE2	0.0300	0.0128	0.0090	0.0060	0.0046	0.980	0.920
IMPUTE4	0.0250	0.0102	0.0062	0.0040	0.0030	0.980	0.940
minimac3	0.0250	0.0100	0.0060	0.0040	0.0030	0.980	0.930
minimac4	0.0250	0.0102	0.0060	0.0040	0.0030	0.980	0.940

*Intervals for the grouping, in which the concordance of SNPs is averaged according to MAF, have the length 0.001, while the intervals for genotype probabilities have the length 0.01*.

Table 4 depicts the mean concordance of the minor allele genotypes with a MAF between 0 and 0.001, which is the rarest category in Figure 4, to showcase the imputation performance for very rare SNPs. PBWT is not included, since the concordance is zero. IMPUTE2 has the lowest concordance for the minor allele genotypes, while Beagle4.1, Beagle5.1, IMPUTE4, minimac3 and minimac4 have similar results with the minimac programs at the top. Phasing with Eagle2.4.1 with a reference panel yields slightly better results for all imputation programs. With the exception of PBWT and IMPUTE2, the concordance ranges between 0.42 and 0.51 for very rare variants.

**Table 4:** Concordance for minor allele genotypes with a MAF between 0 and 0.001.

**Imputation**	**Phasing**				
	**Beagle5.1**	**Eagle2.4.1**	Eagle2.4.1^*^	**SHAPEIT2**	SHAPEIT2^*^
Beagle4.1	0.45	0.46	0.49	0.44	0.48
Beagle5.1	0.42	0.43	0.47	0.40	0.45
IMPUTE2	0.30	0.29	0.34	0.29	0.34
IMPUTE4	0.46	0.47	0.50	0.44	0.49
minimac3	0.48	0.48	0.51	0.46	0.50
minimac4	0.47	0.48	0.51	0.46	0.49

* *Indicates phasing with reference panel*.

Finally, Table 5 lists the computation times for the imputation and phasing process. Since there might be differences in computation time depending on the used processors, we also converted the raw run time into factors. Note, that this is the total run time, so the gain of speed from the parallelization of the chunks is not represented in this table. For imputation, PBWT was the fastest program with a time of 2.67 h, followed by Beagle5.1 with 5.81 h (0.28 h/chunk), while minimac3 was the slowest imputation program requiring 57.29 h (2.73 h/chunk). Within the phasing protocols, SHAPEIT2 using a reference panel was the slowest with a runtime of 24.2 h, while Beagle5.1 was the fastest with a runtime of 3.5 h for both chromosomes.

**Table 5:** Runtime (CPU time) in hours and as factors for imputation and phasing.

**Tools**	**Runtime in hours**		**Runtime as factors**	
	**Imputation**	**Phasing**	**Imputation**	**Phasing**
Beagle5.1	5.81	3.50	2.18	1
Beagle4.1	31.65		11.85	
Eagle2.4.1^*^		4.47		1.28
Eagle2.4.1		4.68		1.33
IMPUTE2	34.57		12.95	
IMPUTE4	32.73		12.26	
minimac3	57.29		21.46	
minimac4	48.69		18.24	
PBWT	2.67		1	
SHAPEIT2		14.07		4.02
SHAPEIT2^*^		24.20		6.91

*For the factors, the fastest tool within imputation and phasing programs was chosen as factor one. For this table, the gain of speed due to parallelization is disregarded, since it is highly dependent on the local machines and chunking. Intel Xeon E5-2680 CPUs were used for phasing and imputation. Phasing tools marked with an asterix indicate the use of a reference panel for phasing*.

## 4. Discussion

To asses the imputation quality of the most recent and commonly used versions of imputation and phasing tools, we compared imputed with known sequenced genotypes in a large German population. In general, the imputation yielded very good results with a mean error rate below 0.005 for most of the tool combinations. The median error rates indicate only small differences in the imputation quality between most of the imputation programs and even less differences between the phasing protocols. The slightly better results of the older versions of Beagle and minimac could be due to adapting the algorithms for data sets larger than ours. The size of our data set might also explain the slight differences between the phasing approaches, and using a reference panel in the phasing process might have a larger impact for smaller data sets.

The PBWT tool's imputation quality was lower than expected, especially considering the high scores shown in Figure 3 which would give a high confidence in PBWT's imputation results, if the true genotypes were unknown. Noteworthy is the median IQS of zero, indicating an imputation quality on the same level as assigning genotypes randomly according to the allele frequencies. The reason for this is most likely that the scores not using true genotypes reward algorithms for imputing SNPs as monomorphic, while the IQS punishes this. This also coincides with the concordance of minor allele genotypes in Figure 4, which might indicate a strong preference to impute the reference allele, even if the MAF is high. The performance of PBWT in our evaluation is in contrast to comparable studies involving the Sanger Imputation Service that is also based on PBWT and yields very good results in terms of imputation quality (Schurz et al., 2019). This suggests that the currently publicly available PBWT program does not include the latest developed tools for PBWT-based imputation yet, and the imputation quality might improve drastically in future releases.

Figure 4 illustrates that even for programs with very high imputation quality, rare variants are likely to be underrepresented in imputed datasets, indicating that SNPs with low MAF should be sequenced and not imputed if they are of particular interest in the study. However, an incorrect imputation of the minor allele genotypes for the rare variants will result in a genotype homozygous with the reference allele, so the loss of information is mostly one-sided, and strong signals might still be detected even with a lower number of correctly imputed minor allele genotypes. A cutoff for SNPs with a low MAF in genotype imputation, if used at all, should comply with the aims of the individual study keeping in mind that rare variants are likely underrepresented, but identifying only a proportion of the rare variants might still outweigh the drawbacks of low concordance rate for those genotypes. Additionally, homozygous genotypes with the reference allele may also be wrongly imputed, possibly leading to false positive associations, however this is assumed to be unlikely in rare variants, and would further emphasize our recommendation of genotyping those areas directly. If direct genotyping for single variants is practically possible, it should be considered for SNPs with a MAF of 0.05 and lower, or 0.03 and lower to ensure a concordance of 95%. In any case, imputation with the minimac programs in combination with Eagle2.4.1 and a phasing reference panel yields the largest proportion of correctly imputed minor allele genotypes. The small drops in the minor allele concordance visible in Figure 4, such as the local minimum around a MAF of 0.04, are likely due to single, difficult to impute SNPs and the small size of the MAF intervals in the figure.

The concordance depending on the genotype probabilities suggests a rather high cutoff of 0.98 to discard possibly incorrectly imputed genotypes if the aim is to only impute genotypes with an error rate of at most 5% on average. We do not neccessarily suggest implementing a threshold this high to accept imputed genotypes in general. Since the concordance rate is not available in a typical imputation setting, this concordance cutoff is more fit to illustrate the rapidly falling imputation quality with lower genotype probabilities. It should be considered for practical use though that the underlying concordance may be considerably lower than the genotype probability suggests. However, since the overall concordance is very high, this suggests that the fraction of genotypes with low genotype probabilities are comparably small. The observation that IMPUTE2 would require a less stringent cutoff than other programs to achieve the same accuracy although the imputation quality is worse might be a result of the newer programs assigning genotype probabilities with more weight on the favored genotype, which coincides with IMPUTE2's results in the Hellinger score.

The higher mean in the Hellinger score for IMPUTE4 indicates that specifically the estimated genotype probabilities of this program are more accurate. This is not a contradiction to minimac3 yielding the lowest mean error rate for best guess genotypes, since the quality measures differ mostly in penalizing deviations to the true genotype according to the output format. The Hellinger score and the SEN score penalize any deviation from a completely unambiguous imputation, even if the best-guess genotype is correct (Roshyara et al., 2014). The Hellinger score using the genotype probabilities is stricter than the SEN score using the dosage for similar reasons, concurring with the results shown in Figure 2.

After our analysis was concluded, both IMPUTE and Beagle released a new version. Beagle5.2^11^ improves speed in phasing, compared to the earlier version, while IMPUTE5 (Rubinacci et al., 2020) improves speed in imputation. While including those newer versions retrospectively would broaden the overview, their release does not detract from our findings in genotype imputation quality, since this seemingly did not improve in the later versions.

The practical speed of phasing and imputation is very dependent on the parallelization and the local available capacities. For imputation with minimac and Beagle, the number of CPUs to be used can be specified directly in the call of the program, while for IMPUTE only chunks can be specified, which would have to be run in parallel by hand or other means such as batchtools. The minimum recommended chunk length, which depends on the chosen program, also needs to be considered for effectiveness.

In general, imputation quality is dependent on the size of the reference panel (Das et al., 2016; Bai et al., 2019). So far, the largest reference panel, to our knowledge the TOPMed panel, currently is only available on an imputation server^12^. IMPUTE5 (Rubinacci et al., 2020) and Beagle5.1 (Browning et al., 2018) are able to handle next-generation reference panels, which might become a factor in choosing an imputation tool in the future. While there is no publication on minimac4 specifically yet, we assume the higher efficiency in both computation and memory used, especially the use of the M3VCF format for reference panels, enables minimac4 to handle larger reference panels than minimac3.

It should be noted that all results of this analysis are based on a real data set of a European population, which is overrepresented in the available genetic data. If other ethnicities are present, other reference panels might be more appropriate (Huang et al., 2009; Li et al., 2009; Schurz et al., 2019), which might be considerably smaller for now. If available reference panels contain less individuals of the target data set's population, the imputation quality is very likely suffering in every aspect. Since we only have European data available, we cannot determine if the loss of quality is larger for one phasing or imputation tool compared to another. Likewise, we cannot necessarily extrapolate our results to data sets containing related individuals. Further, the Illumina chip we emulated is very dense compared to other available genotyping arrays. It follows that the initial situation in this analysis is favorable for good imputation outcomes. Testing imputation quality with less dense genotyping arrays should be considered in further research, since differences in imputation quality between the leading imputation tools might be more pronounced in less advantageous circumstances.

In conclusion, out of the 35 assessed program combinations, 25 yield similarly high quality imputation results. For practical use, switching a familiar program setup likely only has a noticeable impact on the imputation quality if PBWT or IMPUTE2 are used. Minimac3 is the program with both the highest accuracy and the longest runtime. IMPUTE4 yields slightly more accurate genotype probabilities and should be considered if this output format is particularly required for further analysis. Beagle5.1 with its high accuracy, inbuilt phasing function, and fast runtime is arguably the most convenient tool for imputation in this comparison.

## Author Contributions

IK and DG contributed to the conception and design of the study. KS contributed ideas for endpoints of the analysis. Phasing and imputation was conducted by DG and KS. The analysis of the resulting data was performed by KS. The draft was written by KS. All authors contributed to manuscript revision, read, and approved the submitted version.

## Conflict of Interest

The authors declare that the research was conducted in the absence of any commercial or financial relationships that could be construed as a potential conflict of interest.

## Publisher's Note

All claims expressed in this article are solely those of the authors and do not necessarily represent those of their affiliated organizations, or those of the publisher, the editors and the reviewers. Any product that may be evaluated in this article, or claim that may be made by its manufacturer, is not guaranteed or endorsed by the publisher.

## Footnotes

^1^ https://www.sanger.ac.uk/tool/sanger-imputation-service/

^2^ https://ihg4.helmholtz-muenchen.de/cgi-bin/DZHKomics/search.pl

^3^ http://www.gutenberg-gesundheitsstudie.de/ghs/willkommen.html

^4^ http://hchs.hamburg/

^5^ https://www.helmholtz-muenchen.de/kora/index.html

^6^ http://www2.medizin.uni-greifswald.de/cm/fv/ship.html

^7^ https://www.ikmb.uni-kiel.de/

^8^ https://gatk.broadinstitute.org/hc/en-us/articles/360035531612?id=39

^9^ http://www.htslib.org/doc/bcftools.html

^10^ https://github.com/StahlKt/ImputationComparisonPaper2021

^11^ https://faculty.washington.edu/browning/beagle/beagle.html

^12^ https://imputation.biodatacatalyst.nhlbi.nih.gov

## Members of the DZHKomics Resource

For the cohorts: Stephan Felix, Andre Franke, Hugo Katus, Annette Peters, Renate Schnabel, Philipp Wild; for data handling: Jeanette Erdmann, Inke König, Tim Strom; for preparation of samples: Benjamin Meder; for sequencing: Stephan Wolf; for project coordination: Jeanette Erdmann, Heribert Schunkert, Tanja Zeller; for RNA sequencing: Norbert Hübner; for RNA sample preparation: Melanie Waldenberger; for RNA processing: Christian Müller.
