# Supplementary material for: Assessment of Imputation Quality: Comparison of Phasing and Imputation Algorithms in Real Data
Source: Front Genet. 2021 Sep 22;12:724037. doi: 10.3389/fgene.2021.724037 (PMC8493217; doi:10.3389/fgene.2021.724037)
Supplement: Supplementary file 1 [file Data_Sheet_1.PDF]

Supplementary Material

1 TABLES

Table S1. SNP-wise mean of quality measures in the first category for all combinations. For the Hellinger score and the SEN score, only the mean and the minimum of each SNP are included, since the information of the other characteristics is redundant. The phasing tools marked with an asterix indicate the use of a reference panel for phasing.

Imputation	Phasing	Concordance	IQS	Hellinger Minimum	Hellinger Mean	SEN Minimum	SEN Mean
Beagle4.1	Beagle5.1	0.9967	0.7231	0.3274	0.9941	0.8466	0.9993
Beagle4.1	Eagle2.4.1	0.9967	0.7245	0.3305	0.9940	0.8464	0.9993
Beagle4.1	Eagle2.4.1*	0.9968	0.7393	0.3408	0.9943	0.8492	0.9993
Beagle4.1	SHAPEIT2	0.9966	0.7139	0.3169	0.9939	0.8426	0.9992
Beagle4.1	SHAPEIT2*	0.9967	0.7316	0.3301	0.9942	0.8458	0.9993
Beagle5.1	Beagle5.1	0.9967	0.7043	0.3151	0.9941	0.8428	0.9993
Beagle5.1	Eagle2.4.1	0.9966	0.7074	0.3227	0.9941	0.8439	0.9993
Beagle5.1	Eagle2.4.1*	0.9967	0.7256	0.3338	0.9943	0.8471	0.9993
Beagle5.1	SHAPEIT2	0.9965	0.6959	0.3058	0.9939	0.8392	0.9992
Beagle5.1	SHAPEIT2*	0.9967	0.7176	0.3229	0.9942	0.8438	0.9993
IMPUTE2	Beagle5.1	0.9958	0.6381	0.2316	0.9940	0.8099	0.9990
IMPUTE2	Eagle2.4.1	0.9957	0.6360	0.2309	0.9939	0.8093	0.9990
IMPUTE2	Eagle2.4.1*	0.9959	0.6540	0.2455	0.9942	0.8135	0.9990
IMPUTE2	SHAPEIT2	0.9957	0.6318	0.2262	0.9939	0.8079	0.9990
IMPUTE2	SHAPEIT2*	0.9958	0.6540	0.2449	0.9941	0.8131	0.9990
IMPUTE4	Beagle5.1	0.9967	0.7251	0.3607	0.9943	0.8523	0.9992
IMPUTE4	Eagle2.4.1	0.9966	0.7266	0.3634	0.9942	0.8521	0.9992
IMPUTE4	Eagle2.4.1*	0.9967	0.7425	0.3745	0.9945	0.8551	0.9992
IMPUTE4	SHAPEIT2	0.9965	0.7146	0.3456	0.9941	0.8474	0.9992
IMPUTE4	SHAPEIT2*	0.9966	0.7344	0.3621	0.9943	0.8516	0.9992
minimac3	Beagle5.1	0.9969	0.7354	0.3752	0.9933	0.8595	0.9993
minimac3	Eagle2.4.1	0.9968	0.7346	0.3768	0.9932	0.8590	0.9993
minimac3	Eagle2.4.1*	0.9969	0.7495	0.3863	0.9935	0.8615	0.9993
minimac3	SHAPEIT2	0.9968	0.7258	0.3629	0.9930	0.8554	0.9993
minimac3	SHAPEIT2*	0.9969	0.7428	0.3754	0.9933	0.8584	0.9993
minimac4	Beagle5.1	0.9968	0.7337	0.3661	0.9907	0.8579	0.9993
minimac4	Eagle2.4.1	0.9968	0.7332	0.3680	0.9905	0.8576	0.9993
minimac4	Eagle2.4.1*	0.9969	0.7477	0.3776	0.9909	0.8600	0.9993
minimac4	SHAPEIT2	0.9967	0.7245	0.3541	0.9904	0.8539	0.9993
minimac4	SHAPEIT2*	0.9968	0.7414	0.3669	0.9907	0.8570	0.9993
PBWT	Beagle5.1	0.8646	0.0001	0.0001	0.8646	0.5134	0.9313
PBWT	Eagle2.4.1	0.8646	0.0001	0.0001	0.8646	0.5134	0.9313
PBWT	Eagle2.4.1*	0.8646	0.0001	0.0001	0.8646	0.5134	0.9313
PBWT	SHAPEIT2	0.8646	0.0001	0.0001	0.8646	0.5134	0.9313
PBWT	SHAPEIT2*	0.8646	0.0001	0.0001	0.8646	0.5134	0.9313

Table S2. SNP-wise median of the scores in the first category for all combinations. For the Hellinger score and the SEN score, only the mean and the minimum of each SNP are included, since the information of the other characteristics is redundant. The phasing tools marked with an asterix indicate the use of a reference panel for phasing.

Imputation	Phasing	Concordance	IQS	Hellinger Minimum	Hellinger Mean	SEN Minimum	SEN Mean
Beagle4.1	Beagle5.1	0.9991	0.9832	0.1916	0.9984	0.8064	0.9998
Beagle4.1	Eagle2.4.1	0.9991	0.9833	0.1916	0.9984	0.8064	0.9998
Beagle4.1	Eagle2.4.1*	0.9991	0.9871	0.2003	0.9984	0.8108	0.9998
Beagle4.1	SHAPEIT2	0.9991	0.9764	0.1731	0.9983	0.7975	0.9998
Beagle4.1	SHAPEIT2*	0.9991	0.9835	0.1825	0.9984	0.8020	0.9998
Beagle5.1	Beagle5.1	0.9991	0.9725	0.1731	0.9984	0.7930	0.9998
Beagle5.1	Eagle2.4.1	0.9991	0.9746	0.1731	0.9984	0.7975	0.9998
Beagle5.1	Eagle2.4.1*	0.9991	0.9820	0.1916	0.9985	0.8064	0.9998
Beagle5.1	SHAPEIT2	0.9991	0.9634	0.1531	0.9984	0.7884	0.9998
Beagle5.1	SHAPEIT2*	0.9991	0.9763	0.1731	0.9985	0.7975	0.9998
IMPUTE2	Beagle5.1	0.9991	0.8682	0.0000	0.9984	0.7500	0.9998
IMPUTE2	Eagle2.4.1	0.9991	0.8567	0.0000	0.9983	0.7500	0.9998
IMPUTE2	Eagle2.4.1*	0.9991	0.8885	0.0000	0.9984	0.7500	0.9998
IMPUTE2	SHAPEIT2	0.9991	0.8567	0.0000	0.9983	0.7500	0.9998
IMPUTE2	SHAPEIT2*	0.9991	0.8885	0.0000	0.9984	0.7500	0.9998
IMPUTE4	Beagle5.1	0.9991	0.9838	0.2072	0.9986	0.8142	0.9998
IMPUTE4	Eagle2.4.1	0.9991	0.9838	0.2064	0.9985	0.8138	0.9998
IMPUTE4	Eagle2.4.1*	0.9991	0.9887	0.2262	0.9986	0.8240	0.9998
IMPUTE4	SHAPEIT2	0.9991	0.9753	0.1731	0.9985	0.7970	0.9998
IMPUTE4	SHAPEIT2*	0.9991	0.9844	0.1986	0.9986	0.8099	0.9998
minimac3	Beagle5.1	0.9991	0.9875	0.2640	0.9983	0.8440	0.9998
minimac3	Eagle2.4.1	0.9991	0.9871	0.2602	0.9983	0.8420	0.9998
minimac3	Eagle2.4.1*	0.9991	0.9907	0.2772	0.9984	0.8506	0.9998
minimac3	SHAPEIT2	0.9991	0.9824	0.2373	0.9982	0.8298	0.9998
minimac3	SHAPEIT2*	0.9991	0.9878	0.2542	0.9983	0.8388	0.9998
minimac4	Beagle5.1	0.9991	0.9866	0.2512	0.9976	0.8372	0.9998
minimac4	Eagle2.4.1	0.9991	0.9862	0.2482	0.9975	0.8356	0.9998
minimac4	Eagle2.4.1*	0.9991	0.9900	0.2647	0.9977	0.8440	0.9998
minimac4	SHAPEIT2	0.9991	0.9814	0.2254	0.9975	0.8236	0.9998
minimac4	SHAPEIT2*	0.9991	0.9870	0.2420	0.9976	0.8323	0.9998
PBWT	Beagle5.1	0.9948	0.0000	0.0000	0.9948	0.7500	0.9987
PBWT	Eagle2.4.1	0.9948	0.0000	0.0000	0.9948	0.7500	0.9987
PBWT	Eagle2.4.1*	0.9948	0.0000	0.0000	0.9948	0.7500	0.9987
PBWT	SHAPEIT2	0.9948	0.0000	0.0000	0.9948	0.7500	0.9987
PBWT	SHAPEIT2*	0.9948	0.0000	0.0000	0.9948	0.7500	0.9987

Table S3. SNP-wise mean of the quality scores in the second category. Because there is no theoretical boundary for those scores, there are major outliers, which make the mean very hard to interpret. The phasing tools marked with an asterix indicate the use of a reference panel for phasing.

Imputation	Phasing	Beagle R^2	MaCH R^2	IMPUTE Info
Beagle4.1	Beagle5.1	0.9224	0.7591	27.5197
Beagle4.1	Eagle2.4.1	0.6353	0.7604	25.9337
Beagle4.1	Eagle2.4.1*	1.0494	0.7763	22.7302
Beagle4.1	SHAPEIT2	0.8264	0.7527	28.9951
Beagle4.1	SHAPEIT2*	0.6512	0.7703	19.2388
Beagle5.1	Beagle5.1	0.8808	0.7509	16.2175
Beagle5.1	Eagle2.4.1	0.8214	0.7506	15.4275
Beagle5.1	Eagle2.4.1*	0.8189	0.7680	17.5947
Beagle5.1	SHAPEIT2	0.9779	0.7444	12.3199
Beagle5.1	SHAPEIT2*	0.7719	0.7623	13.6177
IMPUTE2	Beagle5.1	1.5873	0.8054	42.2657
IMPUTE2	Eagle2.4.1	1.5581	0.8044	40.5783
IMPUTE2	Eagle2.4.1*	1.9773	0.8148	57.9462
IMPUTE2	SHAPEIT2	1.2482	0.8006	38.4451
IMPUTE2	SHAPEIT2*	2.9383	0.8150	34.8437
IMPUTE4	Beagle5.1	0.0398	0.7634	139.0341
IMPUTE4	Eagle2.4.1	1.4024	0.7645	110.9529
IMPUTE4	Eagle2.4.1*	1.9709	0.7802	190.4401
IMPUTE4	SHAPEIT2	1.1742	0.7568	155.6255
IMPUTE4	SHAPEIT2*	1.0710	0.7747	84.0648
minimac3	Beagle5.1	-0.0603	0.7527	230.9286
minimac3	Eagle2.4.1	1.3124	0.7501	208.5745
minimac3	Eagle2.4.1*	1.0363	0.7660	398.5128
minimac3	SHAPEIT2	1.0638	0.7446	189.1558
minimac3	SHAPEIT2*	1.0853	0.7618	147.0621
minimac4	Beagle5.1	0.3535	0.7500	2.1353
minimac4	Eagle2.4.1	1.0026	0.7472	2.1093
minimac4	Eagle2.4.1*	0.8553	0.7632	2.0521
minimac4	SHAPEIT2	1.4009	0.7420	2.1284
minimac4	SHAPEIT2*	0.6419	0.7588	2.3147
PBWT	Beagle5.1	0.9997	0.9987	0.9994
PBWT	Eagle2.4.1	0.9997	0.9987	0.9994
PBWT	Eagle2.4.1*	0.9997	0.9987	0.9994
PBWT	SHAPEIT2	0.9997	0.9987	0.9994
PBWT	SHAPEIT2*	0.9997	0.9987	0.9994

Table S4. SNP-wise median of the quality scores in the second category for every combination. The phasing tools marked with an asterix indicate the use of a reference panel for phasing.

Imputation	Phasing	Beagle R^2	MaCH R^2	IMPUTE Info
Beagle4.1	Beagle5.1	0.9934	0.9230	0.9369
Beagle4.1	Eagle2.4.1	0.9944	0.9245	0.9391
Beagle4.1	Eagle2.4.1*	0.9901	0.9328	0.9482
Beagle4.1	SHAPEIT2	0.9933	0.9184	0.9315
Beagle4.1	SHAPEIT2*	0.9901	0.9290	0.9437
Beagle5.1	Beagle5.1	0.9986	0.9183	0.9327
Beagle5.1	Eagle2.4.1	0.9991	0.9185	0.9326
Beagle5.1	Eagle2.4.1*	0.9949	0.9272	0.9428
Beagle5.1	SHAPEIT2	0.9990	0.9114	0.9253
Beagle5.1	SHAPEIT2*	0.9950	0.9236	0.9381
IMPUTE2	Beagle5.1	1.0000	0.9572	0.9859
IMPUTE2	Eagle2.4.1	1.0000	0.9565	0.9854
IMPUTE2	Eagle2.4.1*	1.0000	0.9593	0.9882
IMPUTE2	SHAPEIT2	1.0000	0.9556	0.9840
IMPUTE2	SHAPEIT2*	1.0000	0.9594	0.9883
IMPUTE4	Beagle5.1	0.9946	0.9287	0.9425
IMPUTE4	Eagle2.4.1	0.9950	0.9291	0.9434
IMPUTE4	Eagle2.4.1*	0.9930	0.9370	0.9528
IMPUTE4	SHAPEIT2	0.9944	0.9231	0.9362
IMPUTE4	SHAPEIT2*	0.9930	0.9334	0.9478
minimac3	Beagle5.1	0.9888	0.9131	0.9260
minimac3	Eagle2.4.1	0.9893	0.9117	0.9249
minimac3	Eagle2.4.1*	0.9863	0.9219	0.9356
minimac3	SHAPEIT2	0.9890	0.9056	0.9180
minimac3	SHAPEIT2*	0.9862	0.9179	0.9308
minimac4	Beagle5.1	0.9891	0.9101	0.9264
minimac4	Eagle2.4.1	0.9898	0.9086	0.9254
minimac4	Eagle2.4.1*	0.9871	0.9192	0.9363
minimac4	SHAPEIT2	0.9891	0.9025	0.9187
minimac4	SHAPEIT2*	0.9866	0.9151	0.9315
PBWT	Beagle5.1	1.0000	1.0000	1.0000
PBWT	Eagle2.4.1	1.0000	1.0000	1.0000
PBWT	Eagle2.4.1*	1.0000	1.0000	1.0000
PBWT	SHAPEIT2	1.0000	1.0000	1.0000
PBWT	SHAPEIT2*	1.0000	1.0000	1.0000

2 FIGURES

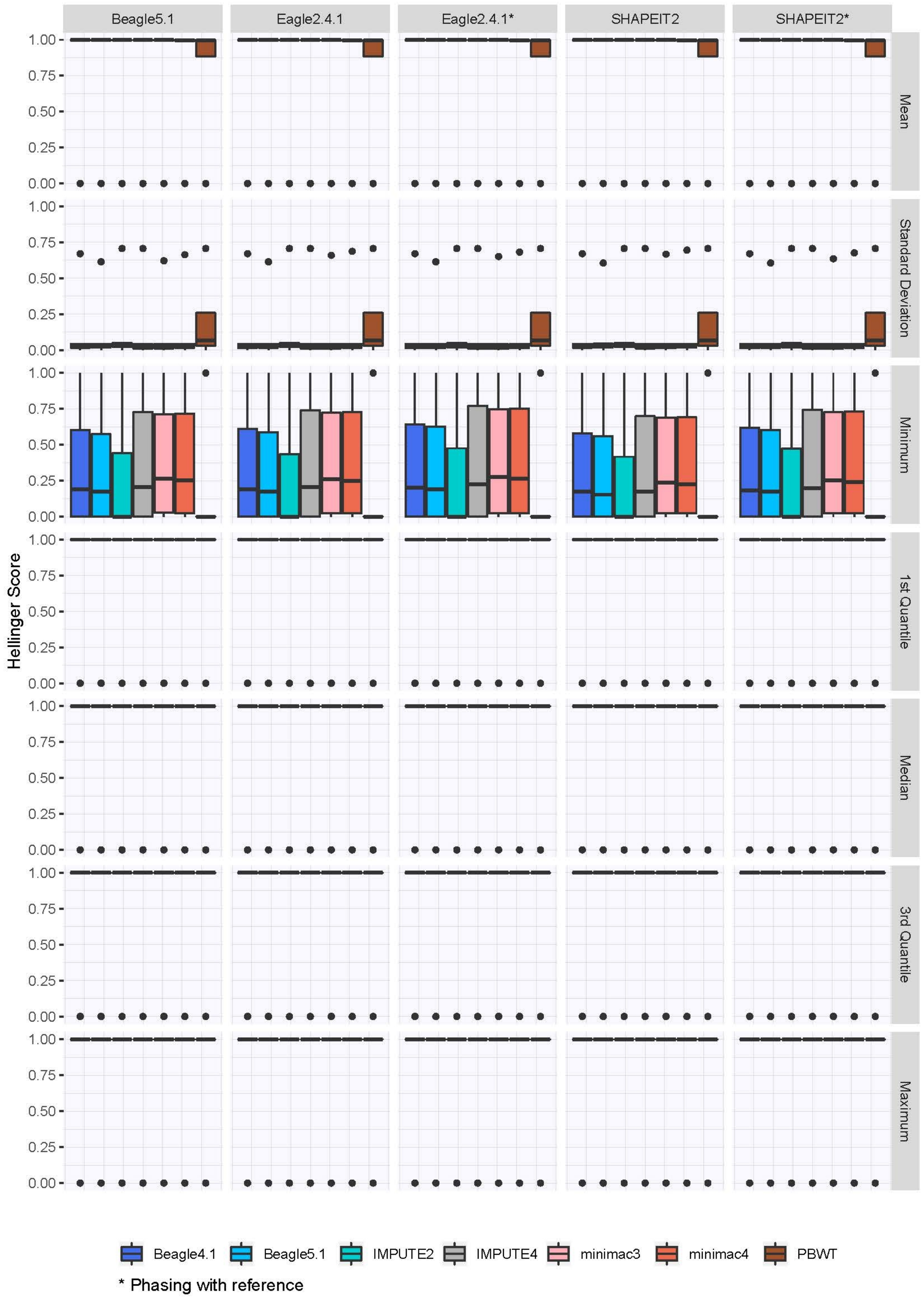

Figure S1. Overview of the Hellinger score. Each row holds the respective descriptive characteristic of the distribution as explained in the main article. The asterix indicates the use of a reference panel for phasing.

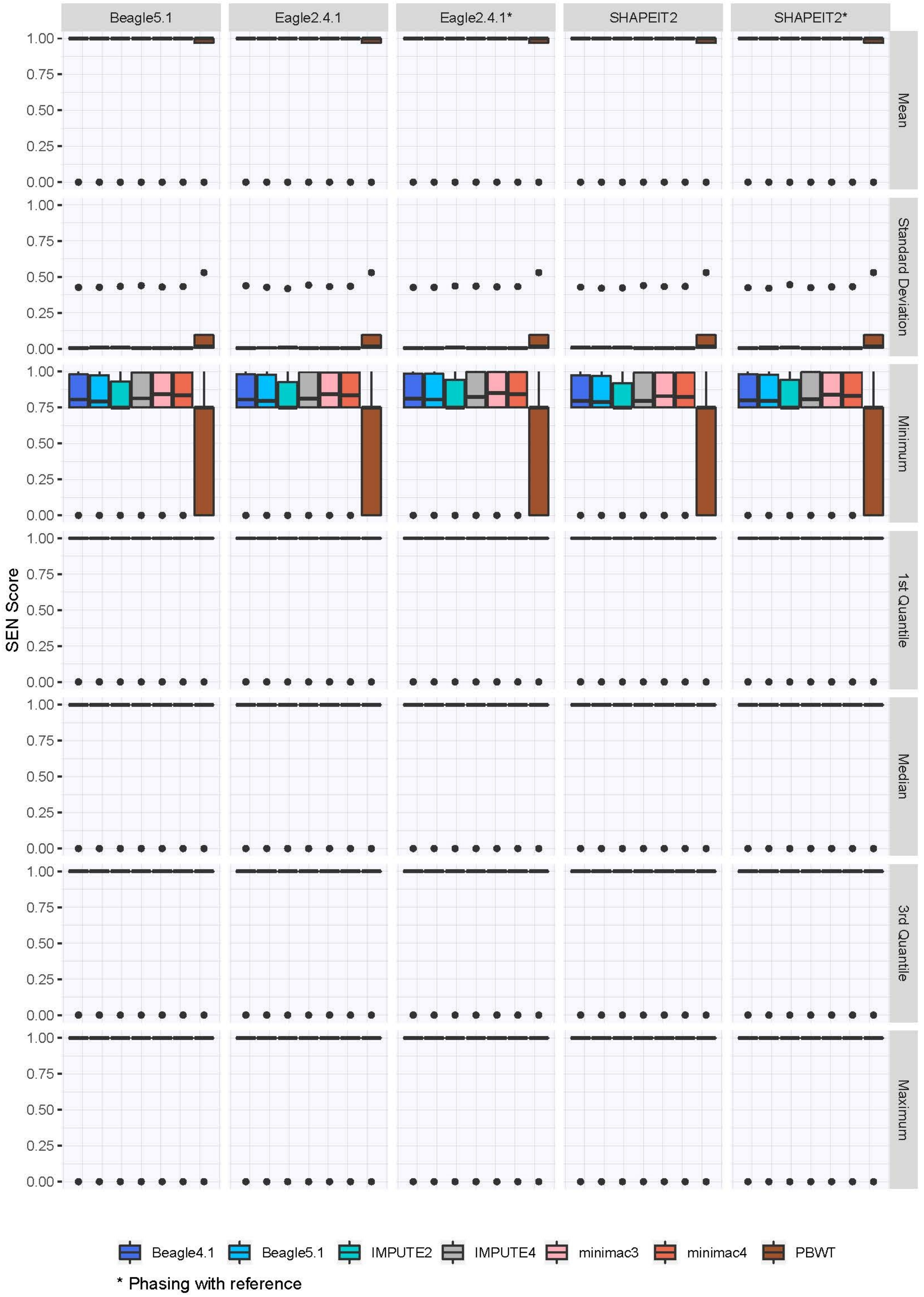

Figure S2. Overview of the SEN score. Each row holds the respective descriptive characteristic of the distribution as explained in the main article for the minimum and the mean. The phasing tools marked with an asterix indicate the use of a reference panel for phasing.

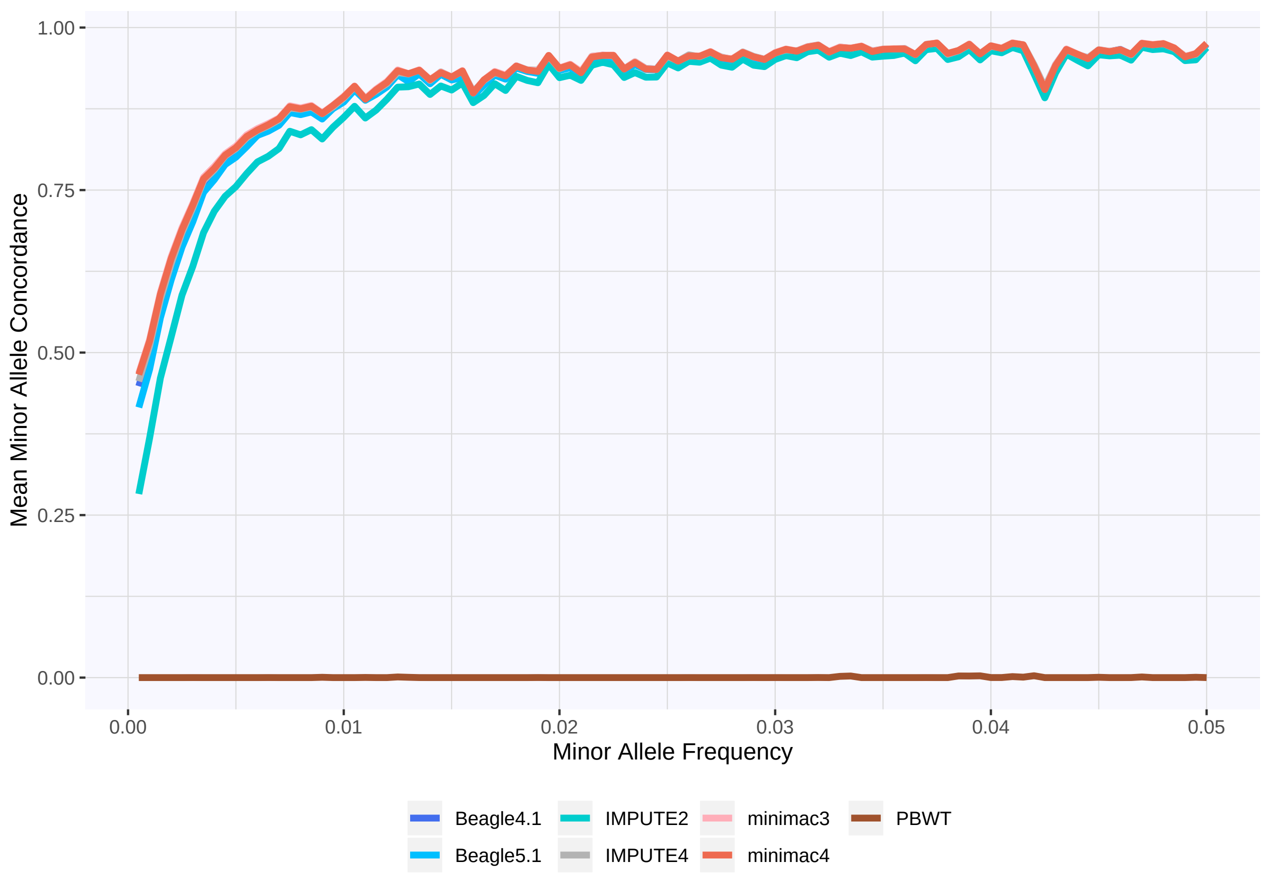

Figure S3. Concordance rate for imputed genotypes with true genotypes not homozygous with the reference allele, depending on MAF, for rare variants with MAF<0.05.

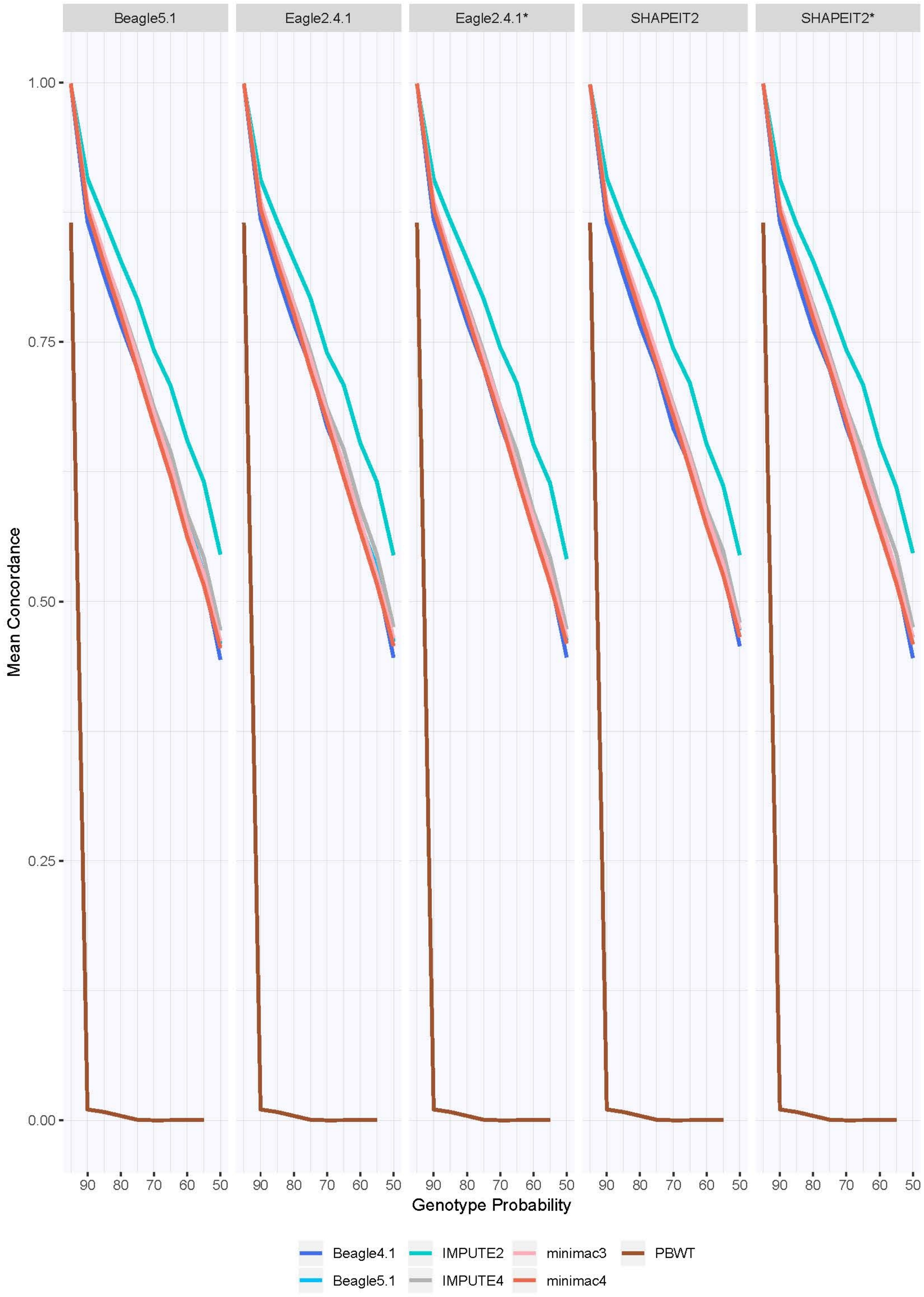

Figure S4. Concordance rate according to genotype probability. For each imputed genotype the genotype probability was taken according to the best guess genotype. In this figure, the interval length of genotype probabilities was set to 0.05 with the lower border fixed to the x axis.
